# COVID-19 and Medical Biotechnology

**Published:** 2020

**Authors:** Nima Rezaei

**Affiliations:** Research Center for Immunodeficiencies, Children’s Medical Center, Tehran University of Medical Sciences, Tehran, Iran

Novel coronavirus disease (COVID-19) pandemic, caused by Severe Acute Respiratory Syndrome Coronavirus 2 (SARSCoV-2), became a global challenge ^1^. The disease which emerged in Wuhan, China, late 2019, has affected more than 6 million individuals in almost all countries and regions, leading to death in more than 350,000 only in a 6-month period (Date: June 1st, 2020). It should be mentioned that SARS-CoV-2 is responsible for the 3rd respiratory syndrome, caused by coronaviruses during last two decades, while SARS-CoV and Middle East Respiratory Syndrome coronavirus (MERS-CoV) were both connected to the emergence of severe respiratory syndromes in 2003 and 2012, respectively ^2^. Meanwhile there is no effective treatment or vaccine for the disease.

Although the pathogenesis of SARS-CoV-2 has not been clearly understood yet, it is a large enveloped virus, similar to other coronaviruses, which contains several proteins including M (membrane), S (spike), E (envelope), and N (nucleocapsid), which are good candidates for targeting ^3^. Among them, S glycoprotein, with two domains of S1 and S2, has been as of interest of recent studies, while it is responsible for invasion and entry into the host cells; the Receptor-Binding Domain (RBD) of S1 interacts with Angiotensin-Converting Enzyme 2 (ACE2) on the cell surface, while the S2 domain is responsible for virus-cell membrane fusion and viral entry with higher affinity ^4^.

Considering the fact that the immune system is affected by the SARS-CoV-2, immune-based treatment, including corticosteroids, monoclonal antibodies against pro-inflammatory cytokines, plasma therapy, and intravenous immunoglobulin was practiced in some patients in a few studies ^5^. However, the efforts should not be limited to such treatments, while novel therapeutic approaches could be considered, using medical biotechnology.

Such pandemic is complex problem, which needs transdisciplinary studies. The development of medical biotechnology to produce pharmaceutical and diagnostic products is a need, which needs close collaboration with other disciplines ^6^. It should be emphasized that it has been clear that coronaviruses know no borders; therefore borderless solution is needed to fight COVID-19 ^7,8^. It is to be hoped that the lessons we learned from SARS-CoV-2, help us to prevent possible pandemic in the near future.
